# Isolated microhematuria in potential kidney donors: evaluating kidney biopsy findings with dipstick urinalysis and urine microscopy results

**DOI:** 10.1093/ckj/sfae371

**Published:** 2024-11-21

**Authors:** Ehab A Hammad, Dalia A Obeid, Dieter C Broering, Yaser Z Shah, Jens G Brockmann, Kris A Marquez, Ahmed M Nazmi, Hassan A Aleid, Hadeel M AlManea, Amira M AlAbassi, Melba A Solomon, Nancy Jacob, Tariq Z Ali

**Affiliations:** Department of Kidney and Pancreas Transplant, Organ Transplant Center of Excellence, King Faisal Specialist Hospital and Research Centre, Riyadh, Saudi Arabia; Transplant Research & Innovation Department, Organ Transplant Centre of Excellence, King Faisal Specialist Hospital and Research Centre, Riyadh, Saudi Arabia; Transplant Research & Innovation Department, Organ Transplant Centre of Excellence, King Faisal Specialist Hospital and Research Centre, Riyadh, Saudi Arabia; College of Medicine, Alfaisal University, Riyadh, Saudi Arabia; Department of Kidney and Pancreas Transplant, Organ Transplant Center of Excellence, King Faisal Specialist Hospital and Research Centre, Riyadh, Saudi Arabia; Department of Kidney and Pancreas Transplant, Organ Transplant Center of Excellence, King Faisal Specialist Hospital and Research Centre, Riyadh, Saudi Arabia; Transplant Research & Innovation Department, Organ Transplant Centre of Excellence, King Faisal Specialist Hospital and Research Centre, Riyadh, Saudi Arabia; Department of Kidney and Pancreas Transplant, Organ Transplant Center of Excellence, King Faisal Specialist Hospital and Research Centre, Riyadh, Saudi Arabia; Department of Kidney and Pancreas Transplant, Organ Transplant Center of Excellence, King Faisal Specialist Hospital and Research Centre, Riyadh, Saudi Arabia; Department of Pathology and Laboratory Medicine, King Faisal Specialist Hospital and Research Center, Riyadh, Saudi Arabia; Abdominal Transplant & Hepatobiliary Surgery Center Department, Organ Transplant Center of Excellence, King Faisal Specialist Hospital and Research Center, Riyadh, Saudi Arabia; Transplant Research & Innovation Department, Organ Transplant Centre of Excellence, King Faisal Specialist Hospital and Research Centre, Riyadh, Saudi Arabia; Transplant Research & Innovation Department, Organ Transplant Centre of Excellence, King Faisal Specialist Hospital and Research Centre, Riyadh, Saudi Arabia; Department of Kidney and Pancreas Transplant, Organ Transplant Center of Excellence, King Faisal Specialist Hospital and Research Centre, Riyadh, Saudi Arabia

**Keywords:** dipstick urinalysis, histopathology, kidney biopsy, kidney donor, urine microscopy

## Abstract

**Background:**

Isolated microhematuria (IMH) can signal hidden glomerular disease, necessitating detailed evaluations for potential kidney donors, including kidney biopsies. The optimal strategy for deciding on kidney biopsies remains unclear. While the British Transplant Society supports dipstick analysis, KDIGO focuses solely on urine microscopy. This study explored the correlation between kidney biopsy outcomes and results from dipstick urinalysis and urine microscopy in potential kidney donors.

**Methods:**

This retrospective study encompassed all potential kidney donors who received kidney biopsies following a positive urine dipstick result for IMH, irrespective of whether red blood cells (RBCs) were found on urine microscopy. We performed sensitivity and specificity analyses to assess the effectiveness of microscopy and dipstick urinalysis in identifying histological abnormalities in the kidney biopsies.

**Results:**

Approximately 49% of potential donors—133 out of 271—who had kidney biopsies due to positive dipstick tests showed negative results in urine microscopy for RBCs. In total, 168 donor candidates, or 62%, had abnormal findings in their biopsies, with nearly half of those diagnosed with immunoglobulin A nephropathy having negative urine microscopy results. Furthermore, 58% of potential donors with negative urine microscopy results—77 out of 133—also exhibited abnormal biopsy findings. The urine microscopy test displayed a sensitivity of 54.2% (95% confidence interval 46.6–61.5) and a specificity of 54.4% (95% confidence interval 44.8–63.7) for detecting abnormal biopsy results.

**Conclusion:**

This study highlighted a significant presence of donors with IMH with underlying glomerular lesions. Using urine microscopy showed limited sensitivity and specificity in identifying abnormal histopathological results. Relying solely on urine microscopy may miss critical pathologies like IgAN in prospective kidney donors. The persistence of IMH during dipstick urinalysis calls for kidney biopsy in potential donors. These findings suggest that our results be incorporated into future global guideline formulations.

KEY LEARNING POINTS **What was known:**

- There is uncertainty regarding the best method for guiding kidney biopsy decisions—urine dipstick or microscopy.
- The British Transplant Society supports dipstick testing, while KDIGO strictly endorses urine microscopy.
- This study explored the relationship between kidney biopsy results and dipstick urinalysis and urine microscopy outcomes in prospective kidney donors.

**This study adds:**

- This study explored the relationship between kidney biopsy results from potential donors and findings from urine dipstick tests and microscopy.
- Urine microscopy, by itself, showed limited sensitivity and specificity in detecting abnormal kidney biopsy results.

**Potential impact:**

- The detection of persistent isolated microhematuria in dipstick urinalysis suggests that kidney biopsy should be considered for potential kidney donors.
- In contrast, urine microscopy alone should not inform decision-making.
- Our findings highlight the need for international organizations to acknowledge these limitations in developing future guidelines, especially regarding evaluating potential kidney donors.

## INTRODUCTION

Kidney donors may face significant long-term risks, evidenced by a higher relative risk of end-stage renal disease (ESRD) among them [1, 2]. The incidence of ESRD in donors primarily stems from immunologically related diseases, particularly in first-degree relatives [2, 3]. Forecasts suggest a significant rise in ESRD cases among kidney donors moving forward [4]. These studies highlight the need for judicious donor selection before kidney donation. Asymptomatic isolated microhematuria (IMH) can suggest the presence of glomerular disease. IMH is frequently observed in the general population [5]. Defining IMH is challenging and poses significant risks. Previously, it was noted that a negative urine dipstick essentially eliminates the possibility of IMH [6]. The urine dipstick test for IMH showed a sensitivity ranging from 91% to 100% and a specificity between 65% and 99% [7].

Nevertheless, urine microscopy is considered a more effective method for detecting and classifying IMH. A prior study determined that examining urine sediments microscopically should steer further IMH investigations, preventing unnecessary costs and invasive procedures [8]. A further study indicates that differences between dipstick results and microscopy continue to exist, even with patient guidance [9]. As a result, urine microscopy is considered unreliable due to the previously mentioned issues. Our publication from 2017 presented data showing that nearly 40% of potential donors with IMH exhibited abnormalities in kidney biopsies. Importantly, this study defined IMH by positive dipstick tests and positive urine microscopy results [10]. As a result, numerous patients showing negative urine microscopy might not have had kidney biopsies.

Weijden *et al*. reported on the outcomes of approximately 700 kidney donors [11], including patients with positive microscopy for IMH. They ruled out donors who only had dipstick analysis and no red blood cells (RBCs) found in microscopy. Importantly, none of these possible donors received a kidney biopsy [11]. As a result, specific donors with glomerular disease might have been allowed to donate. Additionally, another study examined the ≥3 RBCs/high-power field (HPF) threshold, finding that lower levels suggest underlying pathology in biopsies [12]. Consequently, these studies highlighted the ambiguity regarding the best test to determine the need for kidney biopsies in prospective kidney donors.

Discrepancies in international guidelines further complicated the situation. The British guidelines advocate solely for dipstick testing [13]. In contrast, the KDIGO guidelines recommend using urine microscopy to ascertain the presence of IMH [14]. The National Institute for Health and Care Excellence (NICE) guidelines explicitly discourage using urine microscopy to confirm IMH in patients with chronic kidney disease [15]. This variance in recommendations poses difficulties, particularly in the context of kidney donation.

As a leading regional kidney transplant program, we have completed over 5000 kidney transplants. Due to the scarce supply of organs from deceased donors, nearly 80% of these procedures involve living donors. We maintain a low threshold for performing kidney biopsies in patients exhibiting persistent IMH, particularly given our strong dependence on living donors, usually close relatives and often younger individuals. Therefore, we are prepared to correlate urine dipstick results, urine microscopy and pathology findings.

This study investigated the association between potential kidney donors’ kidney biopsy findings and the results obtained from urine dipstick and urine microscopy.

## MATERIALS AND METHODS

This retrospective study examined all prospective kidney donors who had kidney biopsies due to ongoing IMH from November 2015 to February 2024. The institution's Research Ethics Committee (RAC #2 241 143) approved the study, which was conducted according to the Istanbul Declaration.

Every prospective donor at our center received dipstick urinalysis and urine microscopy evaluations on a minimum of two separate occasions and was instructed to provide a morning urine sample. Female donors were generally advised to submit their samples at least a week after their menstrual cycle. Persistent IMH was identified as a positive dipstick result for blood (>1+) in two or more samples. Additional assessments usually comprised urine cultures, renal tract imaging, stone profile analysis, urine cytology and, when necessary, cystoscopy. Kidney biopsy was limited to cases where structural lesions had been excluded. In this study, we included only patients who had undergone a kidney biopsy, excluding those with known structural lesions, albuminuria or proteinuria and those who did not have a biopsy for any other reason. Furthermore, relatives with a family history of Alport syndrome, those with IMH or with positive genetic tests were also excluded from undergoing kidney biopsies.

Our lab performs urinalysis with an automated system. We began with the 77 Elektronika^®^ system and later upgraded to the sophisticated Cobas^®^ 6500 urine analyzer (Roche Diagnostics, Mannheim, Germany). This state-of-the-art system is tailored for urine dipstick and microscopy analysis. The Cobas system offers semiquantitative, qualitative, and quantitative assessments of white blood cells, RBCs, and various other analytes [16]. The dipstick urinalysis usually indicates results from negative, trace positive, 1+, 2+, to as high as 5+. The classification of RBCs/HPF is as follows: 0–5 RBCs/HPF is deemed negative, followed by 6–10, 11–20, 21–30, 31–40, 41–50 and >50 RBCs/HPF.

Potential donors typically underwent kidney biopsies based on positive dipstick tests for IMH, regardless of RBCs in urine microscopy. Biopsy procedures were conducted on an outpatient basis, and individuals were typically discharged approximately 6 h after the procedure.

All kidney biopsy samples were examined according to standard protocols, utilizing hematoxylin and eosin staining, immunofluorescence staining and electron microscopy. Three pathologists who specialize in kidney biopsy evaluations analyzed the specimens. Subsequently, these biopsies were typically reviewed in multidisciplinary meetings that included pathologists and transplant nephrologists.

### Statistical analysis

Data analyses were conducted using R version 4.3.0 (R Core Team, 2024). This study employed inferential and descriptive statistics to explore potential donors’ demographic and clinical profiles. Additionally, sensitivity and specificity analyses assessed the effectiveness of microscopy and dipstick urinalysis in detecting histological abnormalities in kidney biopsies. McNemar's agreement test assessed the concordance of diagnostic tests. Furthermore, univariate and multivariate logistic models evaluated how well the clinical variables of potential donors predicted histological abnormalities. The Kendall rank correlation coefficient examined the relationship between dipstick urinalysis and microscopy results. Statistical significance was set at a threshold of *P* < .05.

## RESULTS

### Donor characteristics and histological findings

The research involved 271 potential kidney donors selected after positive dipstick urinalysis and later had kidney biopsies. Table 1 outlines the histopathology findings and the patient's demographic and clinical information.

**Table 1:** Histopathology results of donor biopsies by patient demographics and clinical characteristics (*N* = 271).

	Biopsy results			
Variable	Overall, *N* = 271^a^	Normal, *N* = 103^a^	Abnormal^c^, *N* = 168^a^	*P*-value^b^
Age	34 (29, 41)	33 (28, 41)	34 (29, 41)	.730
Donor sex				.004^d^
Male	176 (65)	78 (76)	98 (58)	
Female	95 (35)	25 (24)	70 (42)	
Body mass index (kg/m^2^)	26.3 (23.6, 29.4)	26 (23.8, 28.8)	26.7 (23.6, 29.7)	.422
Systolic blood pressure (mmHg)	119 (112, 125)	119 (114, 124)	119 (111, 126)	.803
Diastolic blood pressure (mmHg)	75 (69, 80)	75 (69, 80)	75 (69, 80)	.913
Serum creatinine (μmol/L)	74 (62, 84)	77 (68, 85)	73 (60, 81)	.008^d^
Dipstick urinalysis				0.252
1+	117 (43)	49 (48)	68 (40)	
>1+	154 (57)	54 (52)	100 (60)	
Urine microscopy (RBC/HPF)	0.172			
0–5	133 (49)	56 (54)	77 (46)	
>5	138 (51)	47 (46)	91 (54)	
Donor relationship with recipient				0.198
Sibling	95 (35)	31 (30)	64 (38)	
Offspring	94 (35)	42 (41)	52 (31)	
Other relative	29 (11)	11 (11)	18 (11)	
Parent	28 (10)	7 (6.8)	21 (13)	
Non-related	25 (9.2)	12 (12)	13 (7.7)	
Diagnosis of ESRD				0.361
Unknown	109 (40)	40 (39)	69 (41)	
Diabetes	62 (23)	30 (29)	32 (19)	
Glomerulonephritis	41 (15)	12 (12)	29 (17)	
Hypertension	23 (8.5)	10 (9.7)	13 (7.7)	
Urological	21 (7.7)	6 (5.8)	15 (8.9)	
Others	15 (5.5)	5 (4.9)	10 (6.0)	

a *n* (%); median (IQR).

b Fisher's exact test; Pearson's Chi-squared test; Wilcoxon rank sum test.

c Abnormal biopsy findings account for the glomerular diseases.

d Indicates statistical significance at *P* < .05.

Among our study group, the average age was 34 years, with a majority being male, accounting for 65% (*N* = 176) of potential donors. Nearly half of those potential donors exhibiting IMH on the dipstick urinalysis showed negative results for RBCs in urine microscopy. The most common relationships between potential donors and recipients were among offspring and siblings, making up around 70% (*N* = 189) of the total cases. Almost 40% (*N* = 109) of recipients had been diagnosed with ESRD of unknown cause, while 23% (*N* = 62) had ESRD attributed to diabetes.

Kidney biopsies indicated that about 62% (*N* = 168) of our cohort exhibited glomerular abnormalities. Among the females, nearly 75% (70 out of 95) had these abnormalities, while 55% (98 out of 176) of the males were affected (Chi-square test, *P* = .004). Additionally, the group with abnormal biopsies showed lower pre-biopsy serum creatinine levels than those with normal biopsies. The median serum creatinine was 77 μmol/L [interquartile range (IQR) 68–85] for the normal biopsy group versus 73 μmol/L (IQR 60–81) for the abnormal biopsy group, with a significant difference established by the Wilcoxon rank sum test (*P* = .008). This lower serum creatinine in the abnormal biopsy group stemmed from a gender effect, where men had a median of 81 μmol/L (IQR 74–87) and women had a notably lower median of 58 μmol/L (IQR 53–64). Even after adjusting for gender in the multivariate analysis, this difference was still significant (*P* < .001). However, no other clinical characteristics showed significant correlations with the histopathological findings.

### Donor characteristics and urine microscopy results

In our cohort, 133 individuals (49%) showed negative urine microscopy for RBCs (0–5 RBCs/HPF) despite a positive urine dipstick. Table 2 concisely overviews the urine microscopy results alongside the patients’ demographics and clinical profiles.

**Table 2:** Demographic and clinical characteristics associated with confirmation of IMH through urine microscopy (*N* = 271).

	IMH urine microscopy results			
Patients’ characteristics	Overall, *N* = 271^a^	(0–5) Negative, *N* = 133^a^	(>5) Positive, *N* = 138^a^	*P*-value^b^
Age	34 (29, 41)	32 (28, 40)	35 (29, 43)	.056
Donor sex				.014^c^
Male	176 (65)	96 (72)	80 (58)	
Female	95 (35)	37 (28)	58 (42)	
Body mass index (kg/m^2^)	26.3 (23.6, 29.4)	26.0 (23.0, 29.6)	26.7 (24.0, 29.2)	.447
Histopathological diagnosis				.172
Abnormal	168 (62)	77 (58)	91 (66)	
Normal	103 (38)	56 (42)	47 (34)	
Histopathological findings				.548
Normal	103 (38)	56 (42)	47 (34)	
TBMD	121 (45)	54 (41)	67 (49)	
IgAN	41 (15)	20 (15)	21 (15)	
Other GN	6 (2.2)	3 (2.3)	3 (2.2)	
SBP (mmHg)	119 (112, 125)	119 (111, 124)	119 (113, 126)	.522
DBP (mmHg)	75 (69, 80)	74 (69, 80)	75 (69, 82)	.320
Serum creatinine (mmol/L)	74 (62, 84)	77 (65, 85)	72 (60, 82)	.021^c^
Specific gravity	1.020 (1.012, 1.026)	1.020 (1.012, 1.025)	1.021 (1.010, 1.027)	.341
Diagnosis of ESRD				.063
Unknown	109 (40)	46 (35)	63 (46)	
Diabetes	62 (23)	35 (26)	27 (20)	
Glomerulonephritis	41 (15)	15 (11)	26 (19)	
Hypertension	23 (8.5)	15 (11)	8 (5.8)	
Urological	21 (7.7)	13 (9.8)	8 (5.8)	
Others	15 (5.5)	9 (6.8)	6 (4.3)	
Relationship with recipients				.420
Sibling	95 (35)	46 (35)	49 (36)	
Offspring	94 (35)	45 (34)	49 (36)	
Other relative	29 (11)	19 (14)	10 (7.2)	
Parent	28 (10)	12 (9.0)	16 (12)	
Non-related	25 (9.2)	11 (8.3)	14 (10)	

a *n* (%); median (IQR).

b Fisher's exact test; Pearson's Chi-squared test; Wilcoxon rank sum test.

c Indicates statistical significance at *P* < .05.

Other GN (glomerulonephritis): including, C1q nephropathy = 1, C3 nephropathy = 2 , focal segmental glomerulosclerosis = 1 and membranous nephropathy = 2.

SBP, systolic blood pressure; DBP, diastolic blood pressure.

Females were more likely to have positive urine microscopy than males (72% vs 28%). No statistically significant associations were noted with age, biopsy outcomes, relationship with the potential recipient or ESRD etiology. The study found no significant difference in specific gravity between patients with positive and negative urine microscopy results.

Of the individuals with positive urine microscopy results, 91 (66%) had abnormal biopsy findings. In contrast, 58% of those with negative urine microscopy also showed abnormal biopsy results. Thin basement membrane disease (TBMD) emerged as the most common abnormality in 121 potential donors, with 54 of these donors having negative urine microscopy. A diagnosis of immunoglobulin A nephropathy (IgAN) was made in 41 potential donors, with 20 of them also exhibiting negative urine microscopy. Three patients only had IgA deposition, while the remaining had features indicative of IgAN.

Additionally, urine samples were graded based on the RBCs/HPF identified during microscopy. About 49% (*N* = 133) of the patients showed negative results in urine microscopy (0–5 RBCs/HPF), while 22% (*N* = 61) had 6–10 RBCs/HPF and 16% (*N* = 44) had 11–20 RBCs/HPF. A detailed summary of these microscopic grades and the characteristics of potential donors is presented in Table 3.

**Table 3:** Urine microscopy results stratified by patient demographics and clinical characteristics (*N* = 271).

	0–5, *N* = 133^a^	6–10, *N* = 61^a^	11–20, *N* = 44^a^	21–30, *N* = 18^a^	>30, *N* = 15^a^	*P*-value^b^
Dipstick urinalysis results						
+1	84 (63)	24 (39)	6 (14)	3 (17)	0 (0)	<.001^c^
>+1	49 (37)	37 (61)	38 (86)	15 (83)	15 (100)	
Histopathological findings						
Normal	56 (42)	25 (41)	14 (32)	6 (33)	2 (13)	.662
TBMD	54 (41)	27 (44)	23 (52)	8 (44)	9 (60)	
IgAN	20 (15)	9 (15)	6(14)	3 (17)	3 (20)	
Other GN	3(2.3)	0	1(2.3)	1(5.6)	1(6.7)	
Age	32 (28, 40)	36 (29, 43)	35 (31, 42)	34 (28, 38)	32 (29, 38)	.285
Donor sex						.091
Male	96 (72)	37 (61)	27 (61)	8 (44)	8 (53)	
Female	37 (28)	24 (39)	17 (39)	10 (56)	7 (47)	
Body mass index (kg/m^2^)	26.0 (23.0, 29.6)	26.8 (23.7, 29.4)	27.1 (24.9, 29.8)	26.8 (24.6, 28.3)	24.9 (22.4, 27.8)	.444
SBP (mmHg)	119 (111, 124)	119 (113, 126)	119 (114, 125)	122 (113, 130)	118 (115, 120)	.564
DBP (mmHg)	74 (69, 80)	75 (70, 80)	76 (69, 83)	78 (68, 82)	70 (69, 79)	.599
Pre-donation creatinine (mmol/L)	77 (65, 85)	70 (57, 82)	73 (62, 81)	64 (57, 75)	73 (67, 79)	.148
Donor relationship with recipient						
Sibling	46 (35)	22 (36)	18 (41)	7 (39)	2 (13)	.720
Offspring	45 (34)	22 (36)	14 (32)	7 (39)	6 (40)	
Other relative	19 (14)	6 (9.8)	1 (2.3)	1 (5.6)	2 (13)	
Parent	12 (9.0)	5 (8.2)	7 (16)	2 (11)	2 (13)	
Non-related	11 (8.3)	6 (9.8)	4 (9.1)	1 (5.6)	3 (20)	
Diagnosis of ESRD						
Unknown	46 (35)	23 (38)	26 (59)	8 (44)	6 (40)	.015^c^
Diabetes	35 (26)	10 (16)	10 (23)	3 (17)	4 (27)	
Glomerulonephritis	15 (11.3)	19 (31.2)	4 (9.1)	1 (5.6)	2 (13.3)	
Hypertension	15 (11)	3 (4.9)	2 (4.5)	1 (5.6)	2 (13)	
Urological	13 (9.8)	4 (6.6)	0 (0)	4 (22)	0 (0)	
Others	9 (6.8)	2 (3.3)	2 (4.6)	1 (5.6)	1 (6.7)	

a *n* (%); median (IQR).

b Pearson's Chi-squared test; Kruskal–Wallis rank sum test; Fisher's exact test.

c Indicates statistical significance at *P* < .05.

Other GN (glomerulonephritis): including, C1q nephropathy = 1, C3 nephropathy = 2 , focal segmental glomerulosclerosis = 1 and membranous nephropathy = 2.

SBP, systolic blood pressure; DBP, diastolic blood pressure.

Regarding dipstick urinalysis grades, 63% (*N* = 84) of individuals with 1+ hematuria showed negative results in urine microscopy. In contrast, only 37% (*N* = 49) of those with >1+ hematuria had negative urine microscopy results (Fisher's exact test, *P* < .001). Among the donors with 0–5 RBCs/HPF, which suggests the absence of RBCs, 58% (*N* = 77) exhibited abnormal biopsy results. Notably, there was no significant correlation between the urine microscopy grades and the variables tested, including age and sex.

In conclusion, we performed Kendall's correlation coefficient analysis to evaluate the relationship between microscopy scores (0–5 = 1, 6–10 = 2, 11–20 = 3, 21–30 = 4, >30 = 5) and dipstick urinalysis scores (+1 to +5). The findings indicated a significant correlation (*P < .0001) and a moderate positive correlation coefficient (Tau = 0.44).

### Comparative accuracy of urine microscopy, urinalysis and biopsy results

This study sought to compare the results of microscopy and dipstick urinalysis with biopsy findings; consequently, we analyzed sensitivity and specificity. The main analysis showed that urine microscopy had a sensitivity of 54.2% [95% confidence interval (CI) 46.6–61.5] and a specificity of 54.4% (95% CI 44.8–63.7) in detecting the abnormal biopsy result. The positive predictive value (PPV) was determined to be 66%, suggesting that if the microscopy test is positive, there is a 66% likelihood that the patient has an abnormal biopsy result. Table 4 summarizes the analysis. Our findings reveal a limited diagnostic accuracy of 54.2%, indicating that the relationship between urine microscopy and abnormal biopsy results is statistically significant (*P* = .007).

**Table 4:** Diagnostic approach for identifying glomerular diseases in potential kidney donors with IMH.

Biopsy vs microscopy results			
	Biopsy results		
Microscopy results	Positive	Negative	Total
RBC >5 (positive)	91	47	138
RBC 0–5 (negative)	77	56	133
Total	168	103	271
Parameter		Estimate	Lower, upper 95% CIs
Sensitivity		54.2%	(46.6, 61.5)
Specificity		54.4%	(44.8, 63.7)
Positive predictive value		66.0%	(57.4–73.8)
Negative predictive value		42.1%	(34.1, 50.6)
Diagnostic accuracy		54.2%	(48.3, 60.1)
McNemar's Test *P*-value		.007^a^	

a Indicates statistical significance at *P* ≤ .05.

**Figure 1:**
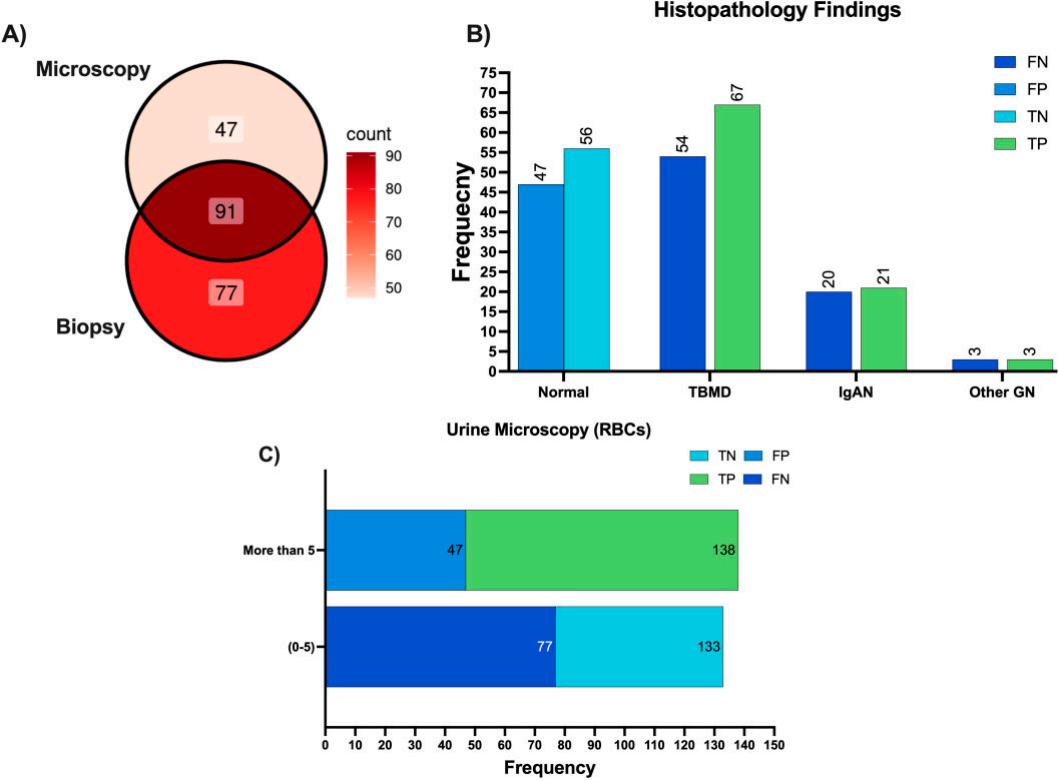
Diagnostic evaluation of urine microscopy and biopsy results in glomerular disease. (**A**) A Venn diagram illustrating the overlap between biopsy results and urine microscopy findings, with a color scale denoting the frequency of cases. The darkest shade represents the highest case counts, while the lightest indicates fewer cases. (**B**) A bar graph depicting the distribution of diagnostic outcomes categorized by histological findings, showing true/false positive and negative results derived from biopsy compared with urine microscopy. (**C**) A bar graph representing the diagnostic performance organized by urine microscopy grades, differentiating between negative (0–5) and positive (>5) results. True/false positive and negative outcomes are compared with biopsy results. Abbreviations: True Positive, True Negative, False Positive, False Negative (FN); Other GN: C1q Nephropathy, C3 Nephropathy, focal segmental glomerulosclerosis, and membranous nephropathy. Bar labels denote the cumulative frequency for each subclass in the figures.

Microscopy and biopsy findings matched in 54% of positive cases (91 out of 168). This left 28% false positives and 17% false negatives (see Fig. 1A). The histopathology results indicated the greatest number of false negatives in the normal biopsy cohort, while false positives were primarily found in patients with TBMD (refer to Fig. 1B). Among donors with 0–5 RBCs/HPF, the false-negative rate was highest compared with biopsy results. Conversely, donors with counts >5 RBCs/HPF showed the most significant true-positive results (Fig. 1C).

**Figure 2:**
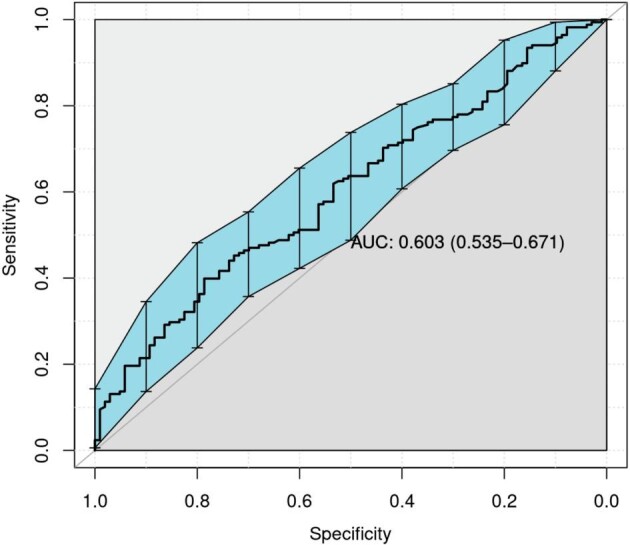
Receiver operating characteristic curve for predicting abnormal histopathological findings using age, sex and microscopy findings. The area under the curve for the model was 0.603 (95% CI 0.535–0.671).

A multivariate binary logistic regression analysis was conducted to predict biopsy abnormalities and microscopy results. Table 5 summarizes the analysis. The model evaluated donor characteristics (age and sex) alongside microscopy findings to predict histopathological abnormalities. Only sex emerged as a significant predictor in the model, which was overall significant with an area under the curve of 0.607 (95% CI 0.54–0.67) (Fig. 2). Furthermore, we examined each variable in a univariate analysis. Again, only sex was significant [female, odds ratio (OR) = 2.20, 95% CI 1.29–3.84], while microscopy (microscopy RBCs >5, OR = 1.40, 95% CI 0.86–2.30) and age (OR = 1.00, 95% CI 0.97–1.03) were not significant in predicting abnormal histopathology.

**Table 5:** Multivariate binary logistic regression model for evaluating biopsy histopathological results and microscopy by donors’ characteristics.

Variables	OR	95% CI	*P*-value*
Age	0.99	0.96, 1.02	.7
Female donor	2.20	1.27, 3.89	.006
Positive microscopy result (RBCs >5)	1.29	0.78, 2.14	.3
Global model *P*-value = .020, AUC = 0.607			

* Indicates statistical significance at *P* < .05.

AUC, area under the curve.

## DISCUSSION

This study found that 62% of potential kidney donors showing IMH on dipstick urinalysis had glomerular lesions in their kidney biopsies. Notably, urine microscopy for RBCs was negative in approximately 50% of these individuals. However, even with negative urine microscopy, 58% of patients exhibited abnormal results in their kidney biopsies. Relying solely on urine microscopy to decide whether to perform kidney biopsies could lead to missing significant conditions, such as IgAN.

Asymptomatic IMH is relatively common in the general population [8, 17, 18]. Further investigation is often needed to identify the cause of IMH. Nonetheless, the reason may still be unclear in many instances. In particular, a prior study found that the cause of IMH was unknown in approximately 80% of the children examined [19]. In addition, IMH indicates underlying severe conditions, such as malignancy [20] or glomerular diseases [21]. A comprehensive study involving more than one million individuals sourced from military service records indicated a higher absolute risk of ESRD in those diagnosed with IMH during regular screenings [22].

Defining IMH poses notable difficulties due to differing opinions on what qualifies as persistent IMH and when additional investigations should occur. Some argue that consistently positive urinalysis (dipstick test) warrants further examination, while others rely on detecting RBCs in urine microscopy as a deciding factor. Notably, a study found that urine dipstick testing is equally effective as urine microscopy in detecting IMH among bladder cancer patients [23]. A different study found that the dipstick test for identifying IMH had sensitivity and specificity rates of 100% and 59%, respectively, compared with urine microscopy [24]. The authors determined that a negative urine dipstick test eliminates the need for additional testing with urine microscopy [24]. Conversely, an earlier study suggested that the choice to carry out IMH investigations should rely on urine microscopy results [8]. They suggested comprehensive urological evaluations for patients with normal-appearing RBCs under microscopy and regular monitoring for those with dysmorphic RBCs without additional invasive tests [8].

Our research indicated that approximately 62% of prospective donors exhibiting IMH on dipstick urinalysis showed signs of glomerular disease upon biopsy. Within this group, 121 were diagnosed with TBMD. Notably, TBMD was identified in some patients with negative RBC findings in urine microscopy (54 out of 121).

TBMD is typically considered a benign condition with an excellent renal prognosis [25]. Nevertheless, some TBMD variants can lead to less ideal outcomes. A prior study indicated that people with seemingly benign hematuria and TBMD might carry heterozygous mutations in the *COL4A3*/*COL4A4* genes, raising the risks of ESRD, hypertension and proteinuria [26]. Additionally, other research has highlighted potential issues with TBMD, suggesting it may not be completely harmless and could lead to adverse renal effects [27, 28]. In the transplant community, kidney donations from donors with TBMD are usually accepted [29]. Our center typically accepts kidney donors with TBMD who are up to 40 years old, following comprehensive counseling. Nevertheless, accurate diagnosis via kidney biopsy is essential for ensuring informed consent, as many patients could be missed if they are not biopsied despite negative urine microscopy results.

The KDIGO [14] and British Transplant Society guidelines generally considered IgAN a contraindication for kidney donation [13]. Among the 271 potential kidney donors in our study, 41 individuals (15%) had IgAN, and 20 showed negative results for RBCs in urine microscopy. Notably, we discovered two patients with C3 nephropathy who also had negative urine microscopy findings. One of these patients presented para-mesangial electron-dense deposits visible on electron microscopy.

If we had depended only on urine microscopy, we might have overlooked multiple patients with underlying IgAN and other glomerular conditions. This highlights the necessity of thorough diagnostic assessments in recognizing and assessing potential kidney donors.

Choosing the right test is essential for diagnosing IMH and assessing whether a kidney biopsy is needed, particularly after excluding other possible causes. Nevertheless, international guidelines still lack agreement, leading to varying recommendations. The KDIGO guidelines suggest that urine microscopy findings should direct further exploration into the underlying cause of IMH [14], a stance influenced by the 2012 guidelines from the American Urology Association (AUA) [30]. The AUA guidelines advocate urine microscopy alone for diagnosing IMH, with positive urine dipstick results not warranting further investigation [30].

Conversely, the British Transplantation Society guidelines recommend using reagent strips and do not endorse urine microscopy for confirming IMH [31]. Our research found that urine microscopy has low sensitivity (54.2%) and specificity (54.4%) for predicting glomerular lesions in kidney biopsies. The urine dipstick detected 62% of donors with glomerular disease, yet 38% still required kidney biopsies, leaving the cause of IMH unclear. Making decisions based on dipstick urinalysis rather than urine microscopy is more beneficial for potential kidney donors, as it can rule out underlying glomerular disease—a risk factor for developing ESRD—and it offers clearer information for both donors and recipients when biopsies reveal normal results or relatively benign pathology.

In our study, the observed sex differences in clinical outcomes and microscopy results may reflect underlying biological and anatomical variations [32]. For instance, lower creatinine levels in females may result from variations in muscle mass and kidney function between genders, affecting outcomes like serum creatinine and biopsy results. Furthermore, previous research has shown that glomerular diseases and their advancement can present differently in each sex, which might explain the differences in microscopic findings [33].

This study has several limitations. It is retrospective, yet we ensured comprehensive data collection and a review of pathology results. We used automated analyzers for urinalysis, which may have inherent limitations. Still, as many centers shift toward automated systems, manual testing is becoming less common. Another limitation is that we did not conduct biopsies on donors with negative urine dipstick results. Thus, we lack data on false-negative results for these tests, as our inclusion criterion required a positive dipstick result.

**Figure 3:**
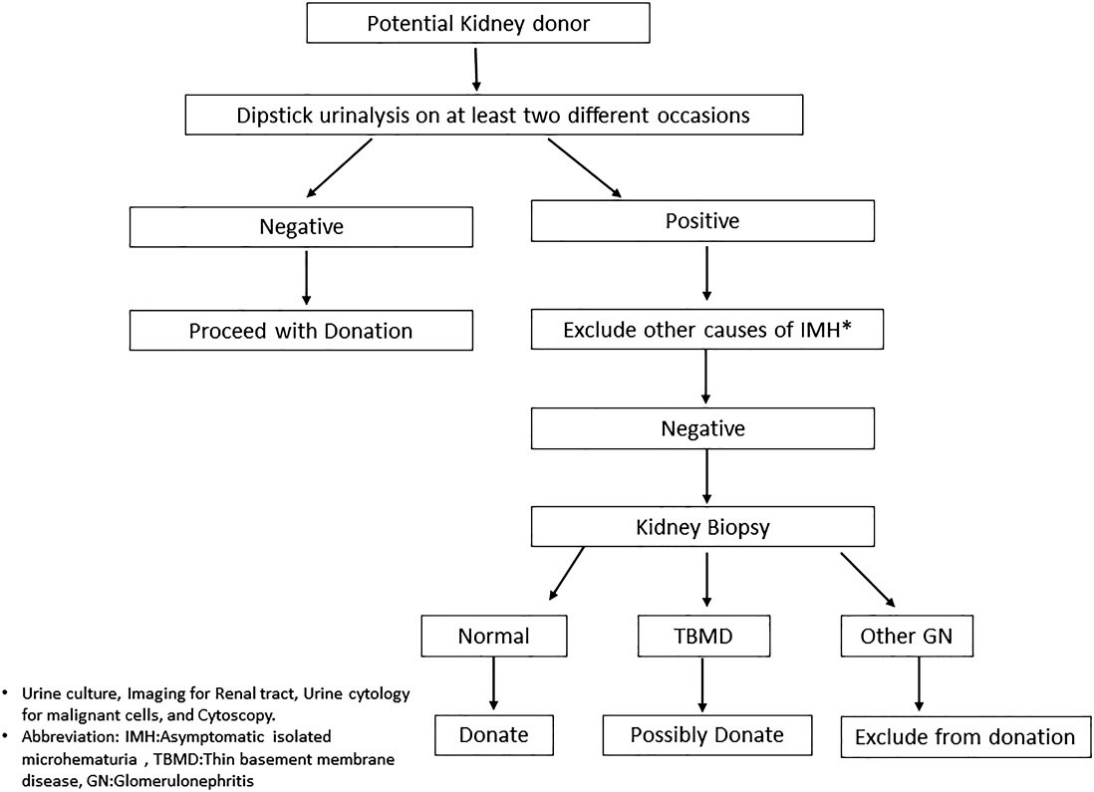
Diagnostic algorithm for potential donors with IMH.

Nonetheless, the chance of a negative dipstick result being associated with positive urine microscopy is exceedingly low [9]. This study focused on potential kidney donors, so the findings should not be applied to the wider healthy population. To our knowledge, prior research has not explored the relationship between urine tests and biopsy outcomes in potential kidney donors, highlighting the limitations of urine microscopy in predicting glomerular diseases. Our results suggest a diagnostic algorithm for potential kidney donors exhibiting microhematuria (see Fig. 3).

In summary, our research showed that many potential donors with IMH had an underlying glomerular issue revealed through kidney biopsies. Urine microscopy is not highly sensitive or specific for identifying abnormal histopathological findings. Thus, if continuous IMH is found on dipstick urinalysis, it suggests the need for kidney biopsy consideration for potential donors, whereas urine microscopy alone should not influence decision-making. Our findings highlight the need for international organizations to address these limitations in the development of future guidelines, especially regarding the evaluation of potential kidney donors.

## Data Availability

The authors will share the raw data supporting the conclusions of this article upon request.
